# Discrepancies in perceptions of well-being: comparing parental and pediatric PROMIS-patient-reported outcomes in Crohn’s disease

**DOI:** 10.1186/s41687-025-00870-9

**Published:** 2025-03-28

**Authors:** Sara Azevedo, Maria Miguel Oliveira, Paulo Jorge Nogueira, Ana Isabel Lopes

**Affiliations:** 1 Gastroenterology Unit, Pediatric Department, Unidade Local de Saúde Santa Maria, Avenida Egas Moniz, Lisbon, 1649-028 Portugal; 2 Faculdade de Medicina, Universidade de Lisboa, Lisbon, Portugal; 3 Área Disciplinar Autónoma de Bioestatística (Laboratório de Biomatemática), Faculdade de Medicina, Universidade de Lisboa, Lisbon, Portugal; 4 Laboratório Associado TERRA, Instituto de Saúde Ambiental, Faculdade de Medicina, Universidade de Lisboa, Lisbon, Portugal; 5 Centro de Investigação, Inovação e Desenvolvimento em Enfermagem de Lisboa (CIDNUR), Escola Superior de Enfermagem de Lisboa, Lisbon, Portugal

**Keywords:** Inflammatory bowel disease, Pediatric Crohn’s disease, Caregivers, Parents, Patient reported outcomes, PROMIS, Health-related quality of life

## Abstract

**Background:**

This study aims to evaluate and compare the perspectives of pediatric Crohn’s disease (CD) patients and their parents/caregivers concerning global physical, emotional, and social health as well as health-related quality of life (HRQQL), using both the Patient-Reported Outcomes Measurement Information System (PROMIS) and the IMPACT III questionnaire.

**Methods:**

In a cross-sectional study, 31 dyads of pediatric CD patients (aged 8–17 years) and their parents/caregivers were recruited from an outpatient Pediatric Gastroenterology Center. Participants completed PROMIS (Global Health, Depressive Symptoms, Anxiety, Meaning and Purpose Pain Interference Life Satisfaction, Peer Relationships, Physical Activity and Fatigue) and IMPACT III measures. Comparative analyses using t-tests and multivariate analyses assessed the impact of demographic factors on score differences. Cohen’s Kappa analysis evaluated the alignment between parent and child perceptions of disease status.

**Results:**

The sample comprised 58% females with a mean age of 15.2 (± 2) years and a mean disease duration of 2.7 (± 2.7) years. Most patients were in disease remission (83.9%) and perceived their disease as better or unchanged in the past 6 months. Concerning PROMIS scores, parents reported significantly lower global health scores (*p* < 0.001) and higher meaning and purpose scores (*p* = 0.029) compared to their children. Parental education and professional status significantly influenced PROMIS score differences. Specifically, mothers with specialized professions showed smaller differences in PROMIS depression and pain interference, although greater differences in PROMIS meaning and purpose, as compared to their respective children’s scores. Fathers with specialized professions demonstrated greater differences in PROMIS anxiety scores but smaller differences in PROMIS life satisfaction scores. A significant misalignment between parent and child subjective perceptions of disease status was observed (*p* = 0.004), suggesting that parents may overestimate symptom severity or underestimate improvements compared to their children’s experiences.

**Conclusion:**

This study highlights the importance of integrating patient and parental perspectives in the clinical management of pediatric CD. The observed discrepancies in disease-related perceptions, influenced by parental educational and professional background, underscore the need for comprehensive assessments to ensure accurate, patient-centered care. For broader generalization, further research should explore these dynamics in newly diagnosed and hospitalized patients.

## Background

Crohn’s disease (CD) is an immune-mediated chronic disease that is widely recognized for its potentially negative impact on the quality of life (QOL), not only for patients but also for their families, who must navigate significant caregiving and emotional burdens. Pediatric CD diagnosis can be challenging, both for the patients and for their families, as they must adjust to a chronic condition, usually severe and progressive. Patients and families experience limitations in everyday activities and school/professional and social activities [1, 19] restrictions. Several pediatric studies have documented that these patients and families experience impaired health-related quality of life (HRQOL) in several physical, psychological, and autonomy domains related to disease activity, requiring specific coping strategies [11, 19]. Also, young IBD patients may experience psychosocial impairment with higher rates of depression and anxiety, further compromising the HRQOL and disease outcome [21].

The younger the patient, the more dependent they are on parental management of the disease, making parental perception a critical factor in treatment adherence and overall well-being. On the other side, pediatric patients should increasingly be able to report symptoms and manage the disease themselves during puberty. The autonomy process in pediatric CD is often compromised due to common pubertal and growth delays, delaying the achievement of adulthood at the expected time [17, 42]. Consequently, these patients may depend on their parents and/or family for extended periods and rely more strongly on family support for disease management. Thus, healthcare providers must increasingly focus on disease management and decision-making for pediatric patients while considering parents’ vital role in successful disease management.

Differences in patient and parental perceptions of disease control (including both management and decision-making) are reasonably well-documented [13, 14, 30, 35, 39] in several pediatric chronic conditions, including IBD. Integration of both perspectives in a so-called “Family-Centered Care Delivery Model” has been recognized as essential for better patient-centered care [39], as the experience of IBD is not uniform across parents and their children as they may perceive the disease differently.

Patient-reported outcomes (PROs) are measures of the outcome of treatments and disease management reported by the patient and/or the caregivers. They highlight the patient’s experience with the disease and assess physical and psychosocial well-being, representing a good model of centered patient care, leading to better-informed healthcare decisions for patients.

Patient- Reported- Outcome-Measurement Information System (PROMIS) is a recent, more specific system of PRO measures developed with funding by the National Institutes of Health (NIH). PROMIS includes measures of global, physical, emotional, and social health. PROMIS includes Pediatric (self-reported items for children age > 8 years) and Proxy measures (parent-reported items for children/adolescents aged 5–17 years) [25, 26, 44], and its reliability and validity have been demonstrated in several pediatric chronic diseases, including pediatric IBD [4, 7, 8]. A cross-sectional study by Arvanitis et al. [4] established a strong correlation between CD disease activity and several pediatric PROMIS measures. Studies comparing patients’ and parents’ perspectives of disease outcomes and HRQOL simultaneously using PROMIS measures are still lacking. They could provide new insight into a more comprehensive patient- and family-centered care [39] as only a few studies compare parents’ and patients’ perspectives on the disease and its overall impact using PROMIS; it is essential to analyze existing literature on similar chronic conditions to draw meaningful comparisons [10, 14, 22, 30].

A study involving children with cancer and their caregivers [37] examined the degree of alignment regarding HRQOL using PROMIS to identify factors associated with better child and caregiver-proxy congruence. This study found that caregivers consistently overestimated symptoms and underestimated mobility relative to the children themselves. Also, including children with cancer, a systematic review was conducted to assess the state of knowledge and agreement regarding the perspectives of the children, their family caregivers, and the healthcare professionals [10]. The authors concluded that there are potential discrepancies between the different perspectives that need to be carefully considered when there are reporters other than the children, highlighting that social-demographic factors and family caregivers’ psychosocial status may influence the perceptions of caregivers.

The study aimed to (1) compare parent-reported and child-reported PROMIS and IMPACT-III scores, (2) evaluate the degree of agreement in disease perception, (3) identify potential demographic factors influencing score differences, and (4) assess the clinical implications of discrepancies in perception. Moreover, we explored the association of differences in PROMIS scores with parental demographic data and disease activity.

## Materials and methods

### Study design and setting

In this cross-sectional study, pediatric CD patients and their parents were consecutively enrolled in an outpatient setting of a single reference center of pediatric gastroenterology during their scheduled appointments over 8 months (January to August 2021).

This study is part of a more extensive study evaluating the clinical utility and responsiveness of the PROMIS pediatric instruments in pediatric CD patients. The main project was developed as two cross-sectional studies. The present study is one of the cross-sectional studies. Another cross-sectional study was designed to investigate PROMIS’s clinical usefulness and applicability by comparing and assessing the correlation between PROMIS and current assessment tools [5]. A longitudinal prospective study with a follow-up period of 18 months, with periodic evaluation every 6 months, was also performed to assess the responsiveness of PROMIS instruments over time in pediatric IBD patients by comparing the responsiveness of PROMIS instruments with that of legacy scales [6].

### Participants

Dyads of caregivers and pediatric CD patients 8–17 years of age who consented to participate were included. Informed assent (< 16 years) and consent (patients 16 years and older and all caregivers of patients) were obtained before enrollment. Patients and/or caregivers who did not sign the written consent, had limitations on verbal or written comprehension of the Portuguese language, or had a recent diagnosis of IBD (duration < 1 month and/or hospitalization) were excluded.

### Variables and data collection

At enrollment, disease-related data were collected from medical records, including CD history, past and current medications, need for hospitalization, and a brief questionnaire covering demographic data for both patients and parents (including parents’ age, level of education, and professional category).

### Data collection

All data were collected during patients’ clinical assessments, and the questionnaires were completed in the waiting room. The study employed structured, researcher-administered questionnaires to minimize missing responses, allowing immediate clarification of doubts and real-time verification of completeness. As a result, no missing data were observed.

### PROMIS measures

Each participant dyad completed nine short forms. PROMIS measures (paper forms):

- Global Health: PROMIS^®^ Parent Proxy Item Bank v1.0 – Global Health – Short Form 7a; PROMIS^®^ Pediatric Item Bank v1.0 – Global Health – Short Form 8a
- Depressive Symptoms: PROMIS^®^ Parent Proxy Item Bank v2.0 – Depressive Symptoms – Short Form 6a; PROMIS^®^ Pediatric Item Bank v2.0 – Depressive Symptoms – Short Form 8a
- Anxiety: PROMIS^®^ Parent Proxy Item Bank v2.0 – Anxiety – Short Form 8a; PROMIS^®^ Pediatric Item Bank v2.0 – Anxiety – Short Form 8a
- Life Satisfaction: PROMIS^®^ Parent Proxy Item Bank v1.0 - Life Satisfaction – Short Form 4a; PROMIS^®^ Pediatric Item Bank v1.0 - Life Satisfaction – Short Form 4a
- Meaning and Purpose: PROMIS^®^ Parent Proxy Item Bank v1.0–Meaning and Purpose– Short Form 4a; PROMIS^®^ Pediatric Item Bank v1.0–Meaning and Purpose – Short Form 4a Physical Activity: PROMIS Pediatric Item Bank v1.0 – Physical Activity – Short Form 4a and PROMIS^®^ Parent Proxy Item Bank v1.0 - Physical Activity – Short Form 4a
- Fatigue: PROMIS^®^ Parent Proxy Item Bank v2.0 – Fatigue – Short Form 10a; PROMIS^®^ Pediatric Item Bank v2.0 – Fatigue – Short Form 10a
- Pain Interference: ROMIS^®^ Parent Proxy Item Bank v2.0 – Pain Interference – Short Form 8a; PROMIS^®^ Pediatric Item Bank v2.0 – Pain Interference – Short Form 8a
- Peer Relationships: PROMIS^®^ Parent Proxy Bank v2.0 – Peer Relationships – Short Form 7a; PROMIS^®^ Pediatric Item Bank v2.0 – Peer Relationships – Short Form 8a

All measures were in Portuguese. Request and permission to translate and use the Portuguese version of PROMIS measures were obtained (translations@HealthMeasures.net). A raw summed score was obtained for each domain and converted into a standardized T-score metric with a mean of 50 and a standard deviation of 10 (11, 12) using the HealthMeasures Scoring Service

Higher scores in any PROMIS domain indicated that more of the domain was being measured. If a PROMIS domain reflects a negative outcome (such as Depressive Symptoms, Anxiety, Pain Interference, and Fatigue), higher scores are indicative of worse well-being; in contrast, higher PROMIS scores in the Global Health, Meaning and Purpose Life Satisfaction, Peer Relationships and Physical Activity domains represent better well-being

### Other variables

Disease phenotype: Classified according to the Paris classification [33].

HRQOL Measurement: The IMPACT III was used to measure HRQOL and applied to both groups. This well-validated and specific measure includes 35 HRQOL-related questions in 6 domains, with scores ranging from 35 to 175, where higher scores indicate better functioning [41].

Disease activity: Assessment using the Pediatric Crohn’s Disease Activity Index PCDAI [23]. Scores range from 0 to 100. A score ≤ 10 indicates inactive disease; scores above this threshold indicate active disease. Patients were divided into two groups according to PCDAI: Inactive disease group (PCDAI 0–10) and mild to moderate active disease (PCDAI 10–35).

Patient and Parents’ perception of disease: A simple semi-quantitative scale was applied regarding the past 6 months (inter-appointment interval): “*feeling better/good*,* feeling the same/not god not bad*,* feeling bad/worsening*” to patients and “*my son/daughter is feeling better/good*,* feeling the same/not god not bad*,* feeling bad/worsening.”* During recruitment and regular clinical appointments, patients were categorized in remissive, mild, moderate, and severe disease based on clinical global assessment (clinical symptoms/signs of active disease, need for treatment escalation, corticosteroid use, hospitalization, treatment compliance, and missed appointments) and PCDAI was assessed.

### Parental education and professional category

Education Level: Classified according to the International Standard Classification of Education (ISCED 2011) (Commission Regulation (EU) No 317/2013). ISCED 0–2: Lower secondary education, ISCED 3–5: Upper secondary education and ISCED > 6: Higher education [43].

Professional Category: Categorized using the Portuguese profession classification, where professions are organized into 9 major groups. The first 3 groups encompass the most differentiated professions [24].

## Statistical methods

Descriptive statistics (mean, median, standard deviation, minimum, and maximum) were computed for each domain of the IMPACT-III and PROMIS scores. Frequencies and percentages were used to summarize categorical variables, such as parental perception of disease status.

To assess the level of agreement between parents and their children, we calculated the intraclass correlation coefficient (ICC) for numerical PROMIS and IMPACT-III scores, using a two-way mixed-effects model with absolute agreement. This approach accounts for potential systematic differences between raters (i.e., parents and children). The ICC value ranged from 0 to 1, where values below 0.5 indicated poor agreement, values between 0.5 and 0.75 indicated moderate agreement, values between 0.75 and 0.9 indicated good agreement, and values above 0.9 indicated excellent agreement.

Conversely, Cohen’s Kappa analysis was applied to assess interrater reliability of categorical disease perceptions reported by parents and children. A linear weighted approach was used to account for the ordinal nature of the data. Kappa values were interpreted as follows: 0–0.20 indicating no agreement, 0.21–0.39 minimal agreement, 0.40–0.59 weak agreement, 0.60–0.79 moderate agreement, 0.80–0.90 strong agreement, and above 0.90 almost perfect agreement. It is important to note that Kappa is influenced by the prevalence of agreement across categories and may not fully capture clinically significant misalignments.

Comparative analyses were conducted to examine the differences between parental and child scores using one sample t-tests, where differences were calculated as the parents’ scores minus the children’s scores. Multivariate analyses were performed to assess the impact of demographic factors (e.g., parental education and employment status) on the differences between PROMIS and IMPACT-III scores. All analysis were conducted using R version 4.3.0 (R Core Team 2023) and statistical significance was assessed at a threshold of α = 0.05.

## Results

### Description of the sample

The study enrolled 31 dyads (all the pediatric patients with CD and their parents’ attending appointments, during the recruitment period) Table 1 summarizes the demographic and clinical characteristics of the pediatric CD patients, and Table 2 details the characteristics of the parents/caregivers.

**Table 1:** Pediatric CD patients’ demographic data and disease-related data

Gender M/F (%) 13/18 (41,9/58.1)	
**Current age**,** years**,** mean (SD)**	15.2 (± 2)
**Level of education (ISCED)** ^**a**^ **n (%)**	ISCED 2: 6 (19.4) ISCED 3–5: 25 (80.6)
**Extracurricular activities n (%)**	9 (29%)
**Duration of disease**,** years mean (SD)**	2.7 (± 2.7)
**Age at diagnosis**,** years**,** mean (SD)**	12.7 (± 3.4)
**Paris age n (%)**: A1a 7 (22.6%) /A1b 24 (77.4%)	
**Paris location n (%)** L1 (Distal 1/3 ileal + limited cecal disease): 1 (3.2%) L2 (Colonic): 3 8 (9.6%) L3 (Ileocolonic): 18 (58.1%) L3L4a (Ileocolonic + Upper discase proximal to Ligament of Treitz):8 (25.9%) L2L4a (Colonic + Upper discase proximal to Ligament of Treitz): 1 (3.2%)	
**Paris phenotype n (%)** B1 (non-stricturing non-penetrating): 27 (87.1%) B2 (Stricturing): 3 (9.7%); B3 (Penetrating): 1 (3. 2%) Perianal disease 5 (16.1%)	
**Paris growth n (%)** G0 (No evidence of growth delay): 27 (87.1%) G1 (Growth delay): 4 (12.9)	
**Current treatment n (%)**	Immunomodulator 15 (48.4%) Immunomodulator + EN ^b^1(3.2%) Immunomodulator + PDN^c^ 2 (6.5%) Anti TNF alfa^d^ 13 (41.9%)
**Need of hospitalization n (%)** ^**e**^	3 (9.7%)
**Need of surgery n (%)** ^**e**^	0 (0%)
**Treatment modifications n (%)** ^**e**^	3 (9.7%
**Need of corticosteroids n (%)** ^**e**^	2 (6.5%)
**Poor compliance to treatment n (%)** ^**e**^	3 (9.7%)
**Mean PCDAI (SD)** ^**f**^	7.5 (16.1)
Inactive disease (PCDAI 0–10): 26 (83.9%)	4.5 (4.2)
Mild to moderate active disease (PCDAI 10–35): 5 (16.1%)	23.0 (9.4)
**Patient’s self-perception of disease n (%)**	
Better	11 (35.5)
Worse	6 (19.3)
No change	14 (45.2)
**Clinical assessment score n (%)**	
Remission	22 (70.9)
Mild	6 (19.4)
Moderate	3 (9.7)
Severe	0

a: ISCED (International Standard Classification of Education): ISCED 0–2: Lower secondary education, ISCED 3–5: Upper secondary education, and ISCED > 6 Higher education

b: enteral nutrition

c: prednisolone

d: anti-Tumoral Necrosis Factor alfa

e: In the prior 6 months

f: Pediatric Crohn’s disease activity index- PCDAI scoring < 10 was considered in remission

SD: Standard Deviation

**Table 2:** Parents’ demographic characteristics

Main Caregiver: Father 10 (33.3%) mother 21 (66.7%)	
**Age of the main caregiver, years**	
Median (Min.; Max.)	44.4 (23.8; 54.5)
Mean (SD)	45.3 (5.8)
**Main Caregiver Level of education (ISCED)** ^**a**^ **n (%)**	
ISCED 0–2	2 (6.5)
ISCED 3–5	17 (54.8)
ISCED ≥ 6	12 (38.7)
**Father Level of education (ISCED)** ^**a**^ **n (%)**	
ISCED 0–2	1 (3.2)
ISCED 3–5	19 (61.3)
ISCED ≥ 6	11 (35.5)
**Mother Level of education (ISCED)** ^**a**^ **n (%)**	
ISCED 0–2	1 (3.2)
ISCED 3–5	18 (58.1)
ISCED ≥ 6	12 (38.7)
**Main Caregiver Professional category** ^**b**^	
Main group 1–3	14 (45.2)
Main group 4–9	17 (54.8)
**Father Professional category** ^**b**^	
Main group 1–3	10 (32.3)
Main group 4–9	21 (66.7)
**Mother Professional category**	
Main group 1–3	13 (41.9)
Main group 4–9	18 (58.1)
**Parent’s perception of disease n (%)** ^**c**^	
Better	17 (54.8)
Worse	6 (19.4)
No change	8 (25.8)

a: ISCED (International Standard Classification of Education): ISCED 0–2: Lower secondary education, ISCED 3–5: Upper secondary education, and ISCED > 6 Higher education

b: Portuguese Professions Classification

c: regarding the past 6 months

SD: Standard Deviation

### Demographic and clinical characteristics of pediatric CD patients

The sample consisted of 58% females with an average age of 15.2 years (± 2).

The mean age at diagnosis was 12.7 years (± 3.4), and the mean disease duration was 2.7 years (± 2.7). Most patients (58.1%) had ileocolonic involvement, and 87.1% exhibited a non-stricturing, non-penetrating disease phenotype without perianal disease (83.8%). No evidence of growth delay was observed in 87.1% of patients; 41.9% of patients were under biological treatment 58.1% were treated with immunomodulators.

The mean Pediatric Crohn’s Disease Activity Index (PCDAI) score was 7.5, with 83.9% (*n* = 26) of the patients in remission (mean PCDAI 4.5) and 16.1% (*n* = 5) with active disease (mean PCDAI 23). Most patients (80.6%) perceived their disease as being better or the same status when compared with the previous 6 months.

### Demographic characteristics of parents/caregivers

The average age of the parents was 45.3 years (± 5.8), with mothers completing most surveys (67.7%). A majority of mothers (71%) and fathers (48.4%) had completed high school and undergraduate studies; 54.8% of parents perceived an improvement in their children’s disease status, while 25.8% reported no changes (Table 2).

### Comparison of parents’ and patients’ PROMIS and IMPACT III scores (Table 3, and 4)

**Table 3:** Global PROMIS and IMPACT scores and differences parents versus patients in PROMIS scores and IMPACT III

Mean (SD) PROMIS scores and differences	Parents/caregivers	Pediatric CD patients	Diff. PROMIS ^a^	*p* ^c^
PROMIS Global Health	37.2 (8.3)	43.2 (7.9)	-5.9 (< 0.001)	*< 0.001*
PROMIS Depressive symptoms	48.9 (11.4)	51.1 (12.7)	-2.2 (0.4)	0.4
PROMIS Anxiety	49.7 (11.4)	50.4 (9.1)	-0.7 (0.7)	0.7
PROMIS Meaning and Purpose	44.0 (9.6)	40.1 (8.1)	3.9 (0.03)	*0.03*
PROMIS Pain interference	50.6 (12)	47.3 (12.6)	3.3 (0.2)	0.2
PROMIS Life satisfaction	43.8 (10.7)	44.1 (10.2)	-0.2 (0.9)	0.9
PROMIS Peer relationships	48.1 (10.5)	50.6 (9.5)	-2.5 (0.2)	0.2
PROMIS Physical Activity	40.6 (9.6)	43.0 (7.2)	-2.4 (0.09)	0.09
PROMIS Fatigue	51.1 (12.8)	52.4 (12.1)	-1.3 (0.5)	0.5
***Mean (SD) IMPACT III Scores and Differences***	***Parents/caregivers***	***Pediatric CD patients***	***Diff IMPACT*** ^***a***^	***p*** ^***c***^
IMPACT III	73.0 (11.6)	73.2 (13.1)	-0.1 (0.9)	0.9
Diff IMPACT III bowel symptoms ^a. b^	-	-	2. (0.3)	0.3
Diff IMPACT III social functioning ^a. b^	-	-	-0.9 (0.6)	0.6
Diff IMPACT III body image ^a. b^	-	-	3.9 (0.2)	0.2
Diff IMPACT III treatment interventions ^a, b^	-	-	0.9 (0.7)	0.8

SD: Standard Deviation

a: Parents score (mean/median) minus (–) their children/adolescent (mean/median) score

b: IMPACT III individual domains

c: Based on t-tests Multivariate analyses

**Table 4:** Agreement between parents’ and patients’ PROMIS and IMPACT-III scores

Measure	Agreement	Interpretation	*p*
Total IMPACT-III	0.67	Moderate	< 0.01
PROMIS Global Health	0.52	Moderate	0.02
PROMIS Depression	0.31	Poor	0.04
PROMIS Anxiety	0.33	Poor	0.04
PROMIS Meaning and Purpose	0.40	Poor	0.01
PROMIS Pain Interference	0.39	Poor	0.01
PROMIS Life Satisfaction	0.58	Moderate	< 0.01
PROMIS Peer Relationships	0.45	Poor	< 0.01
PROMIS Physical Activity	0.57	Moderate	< 0.01
PROMIS Fatigue	0.56	Moderate	< 0.01

Table 3 presents the Global scores and the differences in PROMIS and IMPACT III scores between parents and patients. No significant statistical differences were found between parents and patients’ PROMIS and IMPACT-III scores apart from PROMIS Global Health (*p* < 0.001) and PROMIS Meaning and Purpose (*p* = 0.029) scores, where parents reported lower global health scores and higher meaning and purpose scores, respectively, as compared to their children (Table 3).

Agreement between parents’ and patients’ PROMIS scores was moderate for Global Health, Life Satisfaction, and Fatigue, but poor across the remaining domains (Table 4). For total IMPACT-III scores, we found moderate agreement between parents and patients.

### Impact of demographic factors on PROMIS and IMPACT-III scores (Table 5)

**Table 5:** Impact of demogrphic factors education and profession on PROMIS and IPACT-III scores

PROMIS Measure	More educated mothers^a^		More educated fathers^b^		Less specialized mothers^c^		Less specialized fathers^d^	
	Coefficient (IC 95%)	*p*	Coefficient (IC 95%)	*p*	Coefficient (IC 95%)	*p*	Coefficient (IC 95%)	*p*
Global Health	57.53 (0.1–2.7 x$\:{10}^{4}$)	0.2	0.70 (0.02–25.1)	0.85	0.00 (9.6 x$\:{10}^{-10}$- 94.3)	0.21	4.00 x$\:{10}^{3}$ (0.05–3.4 x$\:{10}^{8}$)	0.15
Depression	0.16$\:(1.3\:\text{x}{10}^{-7}\:-\hspace{0.17em}1.8\:\text{x}{10}^{5}$)	0.79	1.24 x$\:{10}^{-8}$ (1.8 x$\:{10}^{-15}$ − 0.08)	**0.02**	2.81 x$\:\:{10}^{9}$ (6.2–1.3 x$\:{10}^{18}$)	**0.03**	0.38$\:2.3\:\text{x}{10}^{-5}-\:6.4\:\text{x}{10}^{3}$)	0.82
Anxiety	2.30$\:(9.3\:\text{x}{10}^{-7}-\:5.7\:\text{x}{10}^{6}$)	0.91	0.33 (0.00–149.2)	0.72	1.80 x$\:{10}^{6}$ (0.2–1.6 x$\:{10}^{13}$)	0.08	8.69 x$\:{10}^{-7}$ (1.8 x$\:{10}^{-11}$ − 0.04)	**0.01**
Meaning and purpose	835.15 (1–7 x$\:{10}^{5}$)	0.05	0.000 (3.8 x$\:{10}^{-6}\:$- 0.6)	**0.002**	3.95 x$\:{10}^{-8}$ (1.1 x$\:{10}^{-12}$ − 0.001)	**0.001**	2.22 x$\:{10}^{5}$ (4.3–1.1 x$\:{10}^{10}$)	**0.03**
Pain Interference	9.47 x$\:{10}^{-5}$ (7.8x$\:{10}^{-8}\:$- 0.1)	**0.01**	0.26$\:(5.7\:\text{x}{10}^{-5}\:-2\:\text{x}{10}^{4}$)	0.76	7.18 x$\:{10}^{6}$ (4.7–1.1 x$\:{10}^{15})$	**0.03**	0.01$\:(2.2\:\text{x}{10}^{-10}-\:1.6\:\text{x}{10}^{5}$)	0.56
Life Satisfaction	0.18$\:(3.1\:\text{x}{10}^{-5}-\:1\:\text{x}{10}^{3}$)	0.69	0.01 (0.00–0.7)	**0.04**	3.95 x$\:{10}^{-6}$ (5.1 x$\:{10}^{-12}$- 3)	0.69	9.32 x$\:{10}^{6}$ (53 − 1.6 x$\:{10}^{12}$)	**0.01**
Peer Relationships	1.84 (0.001–5.4 x$\:{10}^{3}$)	0.88	0.41 (0.001–160.5)	0.77	0.38$\:(2.3\:\text{x}{10}^{-5}-\:6.4\:\text{x}{10}^{3}$)	0.85	63.83 (0.01–6 x$\:{10}^{5}$)	0.37
Physical Activity	7.26 (0.4–145.4)	0.20	0.39 (0.001–154.2)	0.76	0.05 (4.5 x$\:{10}^{-5}$ − 49.3)	0.39	480.76 (0.4–6.6 x$\:{10}^{5}$)	0.09
Fatigue	1.39 (2.6 x$\:{10}^{-5}$– 7.4 x$\:{10}^{4}$)	0.95	1.48 (0.003–702.7)	0.90	508.53 (0.003–8.1 x$\:{10}^{7}$)	0.31	1.09 (5.8 x$\:{10}^{-5}$– 2 x$\:{10}^{4}$)	0.99

a: Compared with less educated Mothers (ISCED 0–5 lower secondary and upper secondary education versus ISCED > 6 Higher educations)

b: Compared with less educated Fathers (ISCED 0–5 lower secondary and upper secondary education versus ISCED > 6 Higher educations)

c: Compared with more specialized Mothers (Portuguese Professions Classification 0–2 versus 6–9)

d: Compared with more specialized Fathers (Portuguese Professions Classification 0–2 versus 6–9)

Multivariable analysis revealed that parents’ profession and education levels significantly influenced the compared differences in PROMIS and IMPACT-III scores (Table 5).

Mothers’ employment status was associated with differences in PROMIS depression (*p* = 0.032), meaning (*p* = 0.013), and pain (*p* = 0.032) scores, with mothers in specialized professions showing smaller differences in depression and pain scores but greater differences in meaning scores, when comparing to their children scores.

Also, Fathers’ employment status was significantly related to PROMIS anxiety (*p* = 0.011) and life satisfaction (*p* = 0.009) scores, where fathers in specialized professions, when compared to their children scores, demonstrated larger differences in anxiety scores but smaller differences in life satisfaction scores.

Fathers with lower educational levels showed greater differences in PROMIS Meaning and Purpose (*p* = 0.002) and IMPACT social functioning (*p* < 0.001) scores. In comparison, mothers with lower educational levels had significant differences in PROMIS Pain Interference (*p* = 0.011), IMPACT intestinal symptoms (*p* = 0.011), and body image (*p* = 0.015) scores.

### Perception of disease status (Tables 1 and 2)

We found minimal agreement between parents’ and children’s disease perception (Cohen’s kappa = 0.285, *p* = 0.029), suggesting a misalignment between the two groups. Specifically, parents often perceived worsening symptoms when children reported no changes, and parents perceived no changes when children reported improvements.

### Comparison of PROMIS scores in inactive vs. active disease

Analysis revealed that disease activity did not significantly alter the findings; no statistically significant differences were observed between parents’ and patients’ PROMIS scores when comparing inactive versus active disease, according PCDAI (Table 1).

## Discussion

In this study, we comprehensively explored the perspectives of parents and pediatric CD patients regarding the impact of the disease on well-being, general health, and HRQOL, comparing pediatric and by-proxy PROMIS measures. Several studies support the usefulness and responsiveness of PROMIS in various pediatric chronic conditions [15], including pediatric IBD [4, 7, 8]. However, studies using PROMIS to compare parents’ and patients’ perspectives on the disease and its global impact are scarce, although emerging evidence in other chronic pediatric conditions suggests potential discrepancies [14, 22, 30].

The chronic nature of IBD necessitates comprehensive insights from patients and parents, encompassing not only clinical symptoms but also broader physical, psychological, and social dimensions of the disease.

The ideal care model is integrated and patient-centered, involving the patient, their family, and a multidisciplinary team. This approach improves care quality, patient satisfaction, and mental and physical health while reducing healthcare costs [3, 9, 27]. Implementing this model for pediatric patients is challenging, as parents often manage the disease, and pediatric patients may struggle with autonomy in decision-making [17, 42]. Pediatric healthcare providers must prioritize the empowerment of pediatric patients while recognizing parents’ critical role.

Our study found no significant differences between parents and patients reported general health across most domains, except for PROMIS Global Health and Meaning and Purpose scores. Parents perceived lower global health and higher meaning and purpose scores than their children.

This is in agreement with the published evidence, where parents and patients have different experiences with the disease regarding QOL [32, 36, 40], with parents reporting their children as having poorer HRQOL when compared to the patient perspective. Also, in several pediatric chronic conditions, including IBD, adolescent patients and their parents have different perceptions about the disease itself and preferences for disease management and medical treatment [20, 28, 34, 35].

Caregivers of children and adolescents with IBD must navigate the complex task of balancing personal and professional responsibilities with the ongoing demands of disease management, treatment adherence, and adaptation to a new family dynamic [19]. Consequently, it is well-documented that parents of children with chronic conditions such as IBD experience elevated levels of emotional distress and impaired psychosocial functioning [18]. The literature indicates that poor psychosocial functioning in mothers, in particular, is associated with increased depressive symptoms in adolescents and poorer IBD-related health outcomes [19]. This underscores the significant psychosocial gap between parents and children, as parents’ emotional well-being directly influences both their functioning and that of their children.

Moreover, parents often report experiencing work-related difficulties, disruptions to family plans, financial burdens, and marital strain as a result of the IBD diagnosis [2, 16, 18, 19, 29, 31]. These stressors further exacerbate the family’s overall social and emotional functioning. In families that are already characterized by dysfunction or maladjustment, these additional challenges result in a decline in both emotional and behavioral functioning, leading to diminished quality of life HRQOL and an increase in psychological disability for both parents and children [16, 29, 31]. These findings from prior research highlight the psychosocial gap between parents and children in terms of their respective perceptions of meaning and purpose. Parents often experience significant emotional and psychological burdens that may hinder their ability to provide adequate emotional support to their children. This divergence in perspectives may result in misaligned expectations regarding treatment goals, coping strategies, and disease impact, further emphasizing the need for interventions that address both patient and caregiver well-being simultaneously. This divergence is reflected in the heightened emotional distress and negative psychological outcomes observed in both parents and children, as the ongoing chronic illness impacts their individual and collective sense of well-being and life purpose.

The absence of significant differences in most health domains may partially be explained by the long disease duration, where parents and patients might have developed coping skills to adapt to the diagnosis and minimize the disease’s impact [12]. Additionally, most patients were in remission at the time of the survey, which is known to be associated with better patient-reported well-being [4, 7]. The findings, therefore, may be related to the experience of living with a chronic condition and the patient’s family/social support.

Regarding mental health, parents tended to score higher in PROMIS depressive symptoms and anxiety, although no statistical significance was found. A notable discrepancy was observed in PROMIS meaning and purpose and life satisfaction scores, consistent with studies suggesting parents might misinterpret or overestimate their child’s psychological state [12, 28, 31, 32, 34–36, 38, 40]. This highlights the importance of considering both patient and parent perspectives in clinical practice to address potential underreporting or overreporting of symptoms.

An interesting finding was the significant misalignment between parents’ and children’s perceptions of disease status, as indicated by Cohen’s kappa analyses. Parents often perceived a worsening of symptoms when children reported no change and failed to recognize improvements reported by their children. This misalignment could affect disease management and treatment adherence, underscoring the need for healthcare providers to integrate both perspectives into clinical practice. The results, however, must be interpreted with caution as the analyses’ kappa is influenced by the prevalence of agreement and disagreement across categories. Also, as Kappa treats all disagreements the same, the results may fail to account for the clinical relevance of different types of misalignment.

Parents’ educational level and professional status significantly influenced their perception of their children’s health. Mothers with specialized professions showed greater alignment with their children’s reported health outcomes, while fathers’ professional status was linked to differences in mental health assessments. Specifically, lower educational levels in fathers were associated with greater discrepancies in PROMIS Meaning and Purpose and IMPACT social functioning scores. In comparison, lower educational levels in mothers were linked to differences in physical health perceptions. This is consistent with existing literature suggesting that higher educational status and job achievement can enhance understanding and coping mechanisms, potentially leading to more accurate health assessments [13]. It is important to recognize that the confidence intervals for multivariate regression coefficients were extremely wide, suggesting a high degree of uncertainty in the estimated effect size. Although these intervals suggest that associations between score differences and parental sociodemographic characteristics may be statistically significant, the exact magnitude of these effects remains uncertain.

Our results are consistent with previous research showing that parents and patients often have different experiences and perceptions regarding QOL [31, 32, 36, 40], with parents reporting their children has having poorer HRQOL when compared to the patient perspective.

Parents’ more negative view of their child’s HRQOL might influence the care provided, highlighting once more the relevance of incorporating both patient and caregiver perspectives to achieve more patient-centered care [28]. As pediatric patients mature, their involvement in disease management should progressively increase. While parents play a fundamental role in decision-making, a structured transition to patient-led self-management is essential for long-term treatment adherence and autonomy. This transition is particularly important as children with chronic conditions such as Crohn’s disease move into adult care, where independent disease management becomes necessary for optimal outcomes. Healthcare providers should support this shift by integrating both parental guidance and patient perspectives into shared decision-making frameworks. The stress of managing a child’s chronic illness can impact parents’ global health and mood, potentially skewing their perceptions of their child’s well-being [13].

We emphasize the relative disease stability and clinical homogeneity of our patient’s sample (mostly inactive or low activity disease), which might have reinforced the results consistency, allowing the detection of parental vs. patients disagreement, despite disease mostly remissive.

The study, however, has several limitations. The relatively small sample size limits the generalizability of our findings. While sufficient for preliminary analyses, a larger cohort would provide greater statistical power to detect nuanced effects. Additionally, expanding the sample to include newly diagnosed and hospitalized patients may reveal whether disease stage influences parent-child agreement on health outcomes. Additionally, the majority of participants were from middle-class families with higher educational levels, which may not represent the broader population.

Furthermore, the exclusion of recently diagnosed or hospitalized patients reduces the applicability of our results to broader pediatric IBD populations and would allow for subgroup comparisons. Future research should explore these perceptions in newly diagnosed and hospitalized patients, where more pronounced differences might emerge, as newly diagnosed patients and their parents may experience heightened psychological distress and uncertainty, potentially affecting their responses. In contrast, hospitalized patients and their caregivers, who often have more severe diseases or complications, may also report greater impairments in health-related quality of life due to increased symptom burden and functional limitations.

Another limitation is that we did not collect data on parental psychological comorbidities or the use of specific medications, which could influence their perceptions, as the most consistent factors described as associated with worse parent perception of global health and HRQOL is parents’ psychosocial impairment [9, 13, 16, 19, 40]. Furthermore, the predominance of mothers as respondents may limit the generalization of results to both parents.

Despite these limitations, this study further aimed to contribute to the existing literature by highlighting the discrepancies between parent and child perspectives using PROMIS measures. It emphasizes the importance of focusing clinical attention on the pediatric patient’s perspective while simultaneously considering the crucial role of parents in disease management. Furthermore, it reinforces the importance of assessing PROs as an additional tool to the clinical management of pediatric IBD, taking into account that the well-being of patients and their families is an important treatment goal to be achieved, particularly in long-term follow-up. Future research should aim to include more diverse populations and to consider the psychological state of parents to provide a more comprehensive understanding of these dynamics.

## Conclusion

Incorporating both patient and parent perspectives is crucial in managing pediatric CD. The observed discrepancies in perceptions, influenced by parental education and profession, underscore the need for comprehensive assessments to ensure accurate, patient-centered care. Further research should explore these dynamics in diverse clinical settings to enhance the generalizability of findings.

## Data Availability

Not applicable.

## Abbreviations

IBD: Inflammatory Bowel Disease
CD: Crohn’s disease
Pediatric CD: Pediatric Crohn’s disease
PRO’s: Patient Reported Outcomes
PROMIS: Patient- Reported- Outcome-Measurement Information System
HRQOL: Health-Related Quality of Life
QOL: Quality of life
PCDAI: Pediatric Crohn’s Disease Activity Index
